# Extreme Low-Resolution Activity Recognition Using a Super-Resolution-Oriented Generative Adversarial Network

**DOI:** 10.3390/mi12060670

**Published:** 2021-06-08

**Authors:** Mingzheng Hou, Song Liu, Jiliu Zhou, Yi Zhang, Ziliang Feng

**Affiliations:** 1 National Key Laboratory of Fundamental Science on Synthetic Vision, Sichuan University, Chengdu 610065, China; houmingzheng@scu.edu.cn (M.H.); fengziliang@scu.edu.cn (Z.F.); 2 College of Computer Science & College of Software Engineering, Sichuan University, Chengdu 610065, China; 18296875611@163.com

**Keywords:** activity recognition, extreme low-resolution activity recognition, super-resolution, generative network

## Abstract

Activity recognition is a fundamental and crucial task in computer vision. Impressive results have been achieved for activity recognition in high-resolution videos, but for extreme low-resolution videos, which capture the action information at a distance and are vital for preserving privacy, the performance of activity recognition algorithms is far from satisfactory. The reason is that extreme low-resolution (e.g., 12 × 16 pixels) images lack adequate scene and appearance information, which is needed for efficient recognition. To address this problem, we propose a super-resolution-driven generative adversarial network for activity recognition. To fully take advantage of the latent information in low-resolution images, a powerful network module is employed to super-resolve the extremely low-resolution images with a large scale factor. Then, a general activity recognition network is applied to analyze the super-resolved video clips. Extensive experiments on two public benchmarks were conducted to evaluate the effectiveness of our proposed method. The results demonstrate that our method outperforms several state-of-the-art low-resolution activity recognition approaches.

## 1. Introduction

The number of videos created by various recording devices has far surpassed what we can process manually. Therefore, it is crucial to develop intelligent video understanding algorithms for various tasks, such as video recommendation and human activity recognition. Many efforts have been made in the field of activity recognition. Typical methods include the two-stream convolution network [1] and C3D [2]. These approaches assume that the provided videos are high-quality and that video regions of human activities are large enough to model spatiotemporal information. However, in certain situations, such as video surveillance in far-field, where a human is usually very far way from the camera, this assumption is invalid as only low-resolution videos are acquired since the ROI (regions-of-interest) can be extremely tiny in the video frames.

Furthermore, some concerns about privacy protection arise. Increasing numbers of cameras, including security and protection system, wearable devices, and even our cellphones, are recording videos at either public or private places. Even worse is that these recording videos are often stored in the cloud. Concerning our privacy, it is risky to store or upload these videos to remote servers for the reason that they can be leaked or stolen. One possible solution is to transmit the videos with the lowest resolution required for recognition or analysis. However, current methods cannot adapt well to these limitations due to severe changes in extracted features, which raise the challenge of effective activity recognition with extreme low-resolution frames.

In response to this problem, many methods have been proposed. Chen et al. [3] introduced a semi-coupled, filter-sharing two-stream network which utilizes high-resolution videos in the training phase to assist the low-resolution convolutional network (ConvNet) in learning to better distinguish low-resolution features. Based on the observation that training low-resolution videos can benefit from high-resolution data, Xu et al. [4] proposed a fully-coupled two-stream network in which low-resolution videos share all filter parameters with high-resolution videos. Ryoo et al. [5] designed a novel two-stream multi-Siamese network that learns an embedding space shared by low-resolution videos generated with different low-resolution transforms. The aforementioned approaches can be roughly divided into two categories. One is to learn distinguished features for low-resolution data by sharing parameters between high-resolution data and low-resolution videos [3,4,5]. The other is to extract as much latent information from low-resolution images as possible to improve the recognition rate [5]. Due to the utilization of optical flow for temporal information modeling, the computational costs of these methods are high, which impedes their practical application despite the impressive results these methods achieve. Additionally, in semi-coupled and fully-coupled networks [3,4], high-resolution images are only adopted as auxiliary data to assist training, and latent high-resolution information is not fully explored.

Since video resolution has a critical impact on feature extraction, a direct approach is to enhance the video resolution for activity recognition. Recently, learning-based image/video super-resolution (SR) has been broadly studied, and a great number of methods with state-of-the-art performance have been proposed. We have also noticed that a similar idea has been utilized for other topics in low-resolution scenarios and has achieved encouraging results, such as in face recognition, small object detection, and person re-identification [6,7,8].

**Figure 1:**
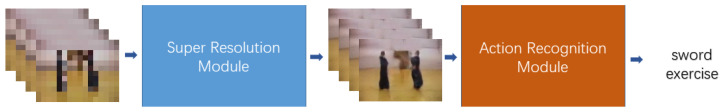
The overview of our approach.

Inspired by this, we propose a super-resolution generative adversarial network for extreme low-resolution activity recognition, which provides a seamless workflow to super-resolve low-resolution images for analyzing human motion. As shown in Figure 1, our approach consists of two modules, namely, a super-resolution module and a spatiotemporal modeling module. Specifically, the super-resolution module can robustly super-resolve high-resolution images from low-resolution images. The spatiotemporal modeling module adopts these generated high-resolution videos as inputs for activity recognition. We must mention that Ugur et al. [9] also proposed a similar method (Prog. DVSR) to ours, which utilizes a progressive generative approach to improve the quality of low-resolution actions followed by a action classifier network. Two main differences exist between both methods: (1) different network structures, including both SR and activity recognition modules are adopted, and (2) Prog. DVSR [9] introduces a weakly trained attention mechanism to help focus on the activity regions in videos, while our approach utilizes long temporal convolution to model the spatiotemporal information in videos.

The main contributions of this paper can be summarized as follows.

(1) We propose an extreme low-resolution activity recognition approach aided by a super-resolution generative adversarial network.
(2) A novel training strategy, called long-range temporal convolution, is used in the recognition module to learn action representations over a long temporal range.
(3) Extensive experiments are conducted, which show that the performance of our approach outperforms several state-of-the-art methods by a large margin despite the fact that we use only RGB images as inputs to avoid the extraction of optical flow.

## 2. Related Work

### 2.1. General Activity Recognition

The existing research in video activity recognition can be broadly categorized into handcrafted and deep learning-based methods. To represent spatiotemporal information of human motion in videos, various handcrafted-based methods, such as space–time interest points (STIP) [10], histogram of optical flow [11], 3D histogram of gradient [12], and SIFT-3D [13] have been proposed. Presently, an improved dense trajectory [14] has been shown to outperform the handcrafted-based approach. Benefiting from the rapid development of deep learning in computer vision, researchers have started to utilize deep models such as VGG [15] and ResNet [16] to represent spatiotemporal information in video clips or image sequences. Karpathy et al. [17] made the first attempt to deploy deep learning for activity recognition. Later, Simonyan and Zisserman [1] proposed a two-stream ConvNet. The two streams of the ConvNet consist of a spatial stream and a temporal stream, which respectively adopt RGB images and optical flow images as inputs. This network obtained a large-margin recognition rate improvement. To model long-range temporal information, Wang [18] introduced a temporal segment network that obtained a high score on two benchmarks: UCF101 [19] and HMDB51 [20]. While these 2D Conv-based methods have achieved impressive results, they face two difficulties. One difficulty is that they cannot effectively model temporal information in videos although optical flows are adopted as inputs. The other difficulty is that extracting optical flow images is time-consuming. These problems were solved by C3D [2], which applies 3D convolutional filters to model spatiotemporal information from short video clips. Later, Carreira [21] inflated 2D convolutional kernels that successfully leveraged parameters pretrained on ImageNet. Qiu et al. [22] further boosted the performance by decomposing 3D convolutional kernels into 2D convolutional kernels in the spatial domain plus 1D convolutional kernels in the temporal domain.

Generally, promising performance has been achieved by these methods to recognize activity in well-prepared videos. However, there are practical demands for low-resolution activity recognition in some specific fields.

### 2.2. Low-Resolution Activity Recognition

To address practical problems, several recent approaches [3,4,5,23] to extreme low-resolution activity have been proposed. These methods can recognize activity to a certain degree in extremely low-resolution (12 × 16 pixels) videos that even humans cannot identify. The key point of these methods is figuring out how to recover or obtain lost visual information with limited pixels and how to fully utilize the information in high-resolution images. Observing that images downsampled from the same image have different pixels, Ryoo et al. [23] proposed the concept of inverse super-resolution (ISR). This method focused on obtaining more information in low-resolution images generated from a single image by learning an optimal set of image transforms. Additionally, to better learn inherent information obtained from multiple low-resolution images, Ryoo et al. [5] introduced a novel multi-Siamese loss. Ryoo’s works are the paradigm for obtaining lost visual information from limited pixels. Another concern is how to utilize high-resolution information. Chen et al. [3] designed a semi-coupled two-stream network in which a low-resolution net shares part filters with a high-resolution net. It employs high-resolution images to assist training. Xu et al. [4] observed that effectively utilizing the information in high-resolution images has a significantly positive impact on the performance improvement of low-resolution recognition. They proposed a fully coupled two-stream network in which high-resolution images are directly adopted as inputs. By utilizing a low-resolution net which shares all convolutional filters with a high-resolution net, the performance of the fully coupled two-stream network is marginally outperformed other methods. In addition, Ugur et al. [9] built a natural low-resolution benchmark TinyVIRAT (https://www.crcv.ucf.edu/tiny-actions-challenge-cvpr2021/, accessed on 3 July 2020) and proposed a novel method which utilizes a progressive generative approach to improve the quality of low-resolution actions.

Revisiting the approaches [5,23] proposed by Ryoo et al., the significance of recovering or obtaining lost visual information from limited pixels is repeatedly highlighted. From these coupled series methods [3,4], we find that utilizing information in high-resolution images is equally important. However, Ryoo et al. did not leverage information in high-resolution images. A coupled network [3,4] adopts only high-resolution images as inputs to assist in training distinguished features, while low-resolution images are not actually enhanced by the useful information in high-resolution images. Therefore, we introduce a super-resolution module that can simultaneously and effectively recover lost visual information and utilize high-resolution information to enhance low-resolution information.

### 2.3. Super-Resolution in Other Low-Resolution Recognition Field

On the other hand, many works [6,7,8] in other low-resolution fields, such as low-resolution face verification, small object detection, and low-resolution person re-identification, employ a super-resolution method to address the low-resolution problem and have all achieved impressive results. Ataer-Cansizoglu et al. [6] proposed a deep learning approach based on identity-preserving super-resolution for very low-resolution face verification. Bai et al. [7] designed an end-to-end multitask generative adversarial network for small object detection. To address the low-resolution and scale mismatching problem in person re-identification, Wang [8] proposed a cascade super-resolution generative adversarial network.

## 3. The Approach

As shown in Figure 1, in this section we describe, in detail, our approach for extreme low-resolution activity recognition. The basic architecture of our super-resolution module, which adopts a generative adversarial network that can robustly recover images with limited pixels, is discussed first. To utilize information in high-resolution images, we also hold the assumption that high-resolution training videos are available. Then, we introduce the basic architecture of our activity recognition module, which employs a 3D residual convolutional network as a spatiotemporal representation model. Finally, a training strategy, called long-range temporal convolution, will be introduced.

### 3.1. Super-Resolution Module

**Figure 2:**
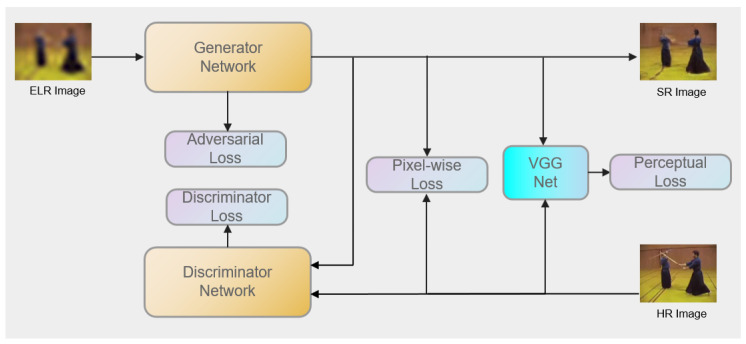
The general architecture of our super-resolution module. ELR denotes extreme low resolution, and SR and HR represent super-resolution and high resolution, respectively.

Similar to most prior works [3,4,23], we assume that in the training phase, we have high-resolution videos. Unlike semi-coupled [3] and fully coupled [4] networks that take high-resolution images as inputs to learn distinguished features, we recover low-resolution images via a generative adversarial network to enhance low-resolution features. Figure 2 shows the general architecture of our super-resolution module.

#### 3.1.1. Generative Adversarial Network

Since generative adversarial network (GAN) [24] was proposed, its strong performance in generating life-like images has impressed us. GANs optimize the generator and discriminator, in turn, via an adversarial process, which enables the generator to achieve an optimal state. The loss of a GAN can be formulated as follows:

(1) $$L_{GAN}=E_{x∼P_{data}\left(x\right)}\left[\log D_{\theta }\right]+E_{z∼P_{z}\left(z\right)}\left[\log\left(1-D_{\theta }\left(G_{w}\left(z\right)\right)\right)\right]$$

where *x* represents the real data, *z* denotes the random noise, and $D_{\theta }$ and $G_{w}$ stand for the discriminator and generator, respectively. The adversarial process between the discriminator and generator can be formulated to

(2) $$\arg\min_{G}\max_{D}L_{GAN}\left(G,D\right)$$

Our goal is simultaneously recovering low-resolution images and obtaining distinguishable features for activity recognition. It is difficult to obtain lost visual information from such limited pixels (12 × 16). More importantly, unlike other super-resolution tasks [25,26] that are focused on reconstructing images without losing details, we concentrate on recovering lost information that can contribute to recognition. Many studies [2,21,22] have confirmed that capturing the motion of humans to model spatiotemporal information is vital for activity recognition. Therefore, the lost information we want to recover from limited pixels is clear silhouettes of humans and objects, which can be used to model the motion of humans. In summary, the proposed GAN should have the ability to deal with large downscale factors and to roughly restore the outline of humans. Inspired by prior attempts [25,26,27] in super-resolution with a large scale factor (×8), we adopt the unique architecture of a generator that can effectively deal with a large scale factor in SDSR [27] and the relativistic discriminator used in ESRGAN [26].

#### 3.1.2. Network Architecture

**Figure 3:**
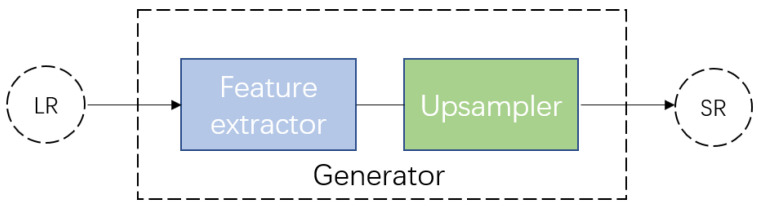
The architecture of our generator.

**Figure 4:**
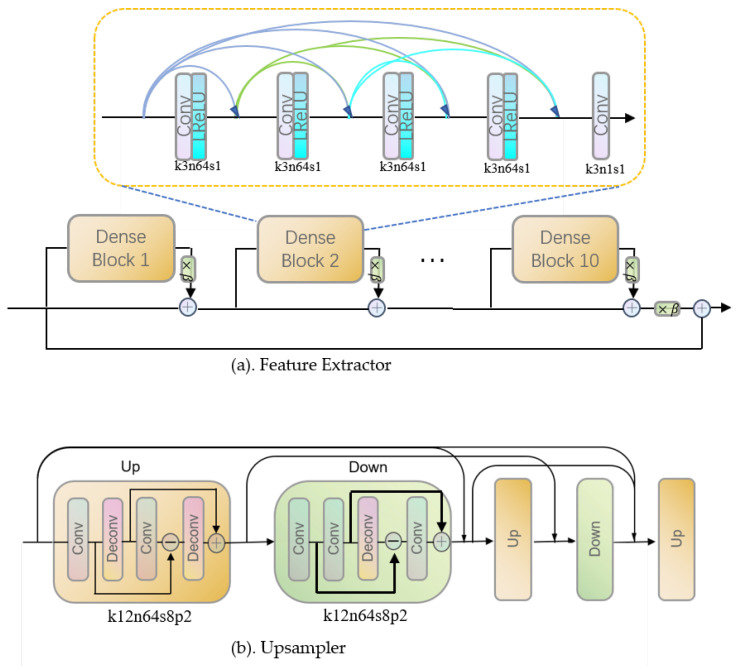
The architecture of the feature extractor and upsampler. Illustration of the (**a**) feature extractor and (**b**) upsampler. β is the residual scaling parameter, which is set to 0.2 k and denotes the kernel size, n represents the number of filters, s is the size of the stride, and p is the size of padding. In (**b**) the conv and deconv share the same numbers of kernel size, features, stride, and padding.

Our generator consists of a feature extractor and an upsampler. Figure 3 demonstrates the general architecture of our generator, and Figure 4 illustrates, in detail, the architectures of the feature extractor and upsampler. In particular, the feature extractor in the generator we used in SDSR [27] adopts the dense deep back-projection network (D-DBPN) [28] as the backbone and improves the ability to extract features from extreme low-resolution images by utilizing the residual in the residual dense block (RRDB) proposed by Wang et al. [26]. The number of RRDB blocks in our feature extractor is set to 10. To learn effective mapping from extreme low-resolution images to high-resolution images, the unique architecture of the upsampler in SDSR [27] is employed. In the upsampler, the features extracted from extreme low-resolution images are upscaled and downscaled alternatively with deep back-projection layers. Specifically, the extracted features are upscaled three times and downscaled two times using the architecture illustrated in Figure 4b. Borrowing the idea from ESRGAN [27], we adopt the relativistic discriminator [29] to determine whether the high-resolution label is more realistic than the generated image.

#### 3.1.3. The Loss Function for the Super-Resolution Module

The loss function is critical for the performance of our super-resolution module. Generally, the key component of a GAN’s loss is MSE. We additionally introduce the SRGAN adversarial loss and VGG loss to measure the perception similarity between generated images and ground truth. In the following, the details of the MSE loss, adversarial loss, and perception loss-based VGG network are described.

**MSE loss.** Pixel-wise MSE loss can be computed using the following equation:

(3) $$L_{MSE}=\frac{1}{r^{2}WH}\sum_{x=1}^{rW}\sum_{y=1}^{rH}{\left(I_{x,y}^{HR}-G_{\theta }{\left(I^{LR}\right)}_{x,y}\right)}^{2}$$

where $I^{HR}$ and $I^{LR}$ respectively represent the high-resolution image and low-resolution image. rW and rH is the size of high-resolution image, where *r* is the factor of downsampling.

**Perception loss.** Perception loss is usually used to measure the similarity in feature space, which has proven efficient for SR. Here, we also introduce perception loss to improve the SR performance and a pretrained VGG-19 network is adopted to extract the features from the first 12 convolution layers. We use Φ to represent VGG network extracting features. The perception loss is calculated as follows

(4) $$L_{perc}=\frac{1}{WH}\sum_{x=1}^{W}\sum_{y=1}^{H}{\left(\Phi {\left(I^{HR}\right)}_{x,y}-\Phi {\left(G_{\theta }\left(I^{LR}\right)\right)}_{x,y}\right)}^{2}$$

where *W* and *H* respectively denote the dimensions of the feature maps extracted by VGG network.

**Adversarial loss.** In addition, adversarial loss is used and it can be calculated as follows:

(5) $$L_{adv}=-\log D_{\theta }\left(G_{\theta }\left(I^{LR}\right)\right)$$

where $D_{\theta }\left(G_{\theta }\left(I^{LR}\right)\right)$ is adopted to distinguish the super-resolved image $G_{\theta }\left(I^{LR}\right)$ from the ground truth image. Finally, the total loss is obtained by combining the MSE loss, perception loss, and adversarial loss as follows:

(6) $$L_{gen}=L_{MSE}+\alpha L_{perc}+\gamma L_{adv}$$

where α and γ are weights trading off the different terms. We set weights α=0.006,γ=0.001 in this paper.

### 3.2. Activity Recognition Module

Formally, we assume that we are given extreme low-resolution videos with *L* frames. A random frame $L_{1}$ is then selected as a temporal start point to generate a video clip $\left\{L_{1},L_{2},L_{3},\ldots ,L_{k}\right\}$. Our goal is to recognize the activity in such extreme low-resolution videos. The process can be represented as follows:

(7) $$score=H(F\left(G\left(L_{1};w\right),G\left(L_{2};w\right),\ldots ,G\left(L_{k};w\right)\right))$$

where *G* is the generator of our super-resolution module, and *w* represents its parameters. *F* can be an arbitrary end-to-end activity recognition model. Different from prior works [3,4,5] that employed a two-stream network adopting optical flow as inputs, a residual 3D convolutional network [30] is selected as *F* due to its powerful ability to model spatiotemporal information and avoid precomputing optical flow. Based on the output of model *F*, the probability of each activity class will be computed by the prediction function *H*. Here, we adopt the softmax function for *H*.

**Figure 5:**
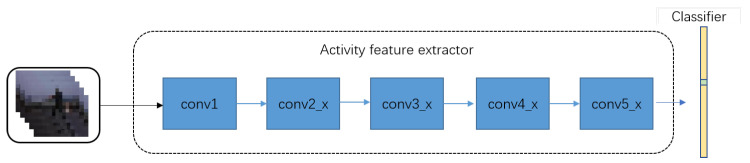
The architecture of our activity recognition module.

**Figure 6:**
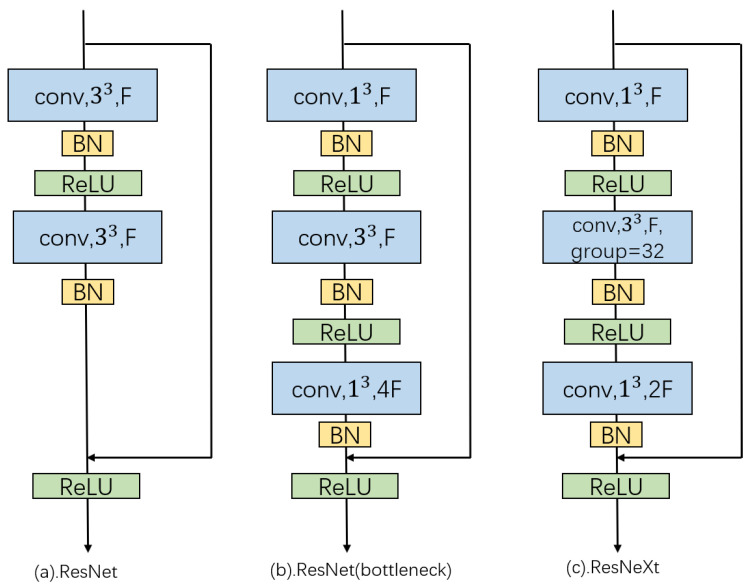
Block of each architecture. conv and $3^{3}$ denote a 3 × 3 × 3 convolutional filter, while F and group is the number of feature channels and groups of group convolution, respectively.

Specifically, the architecture of activity recognition module is shown in Figure 5 and details of each part are illustrated in Table 1. Our recognizer consists of 5 convolutional parts of which the 1st part includes 64 7 × 7 × 7 convolutional filters and the remaining parts are composed of ResNeXt blocks. The series of ResNet block have a strong power on extracting feature and can alleviate the problem of gradient vanishing. Figure 6 depicts the block architecture of ResNet series in which ResNeXt is adopted since its group convolution further eases training and improves performance.

Formally, we use cross entropy loss to train the activity recognition module, and the loss function can be given as follows:

(8) $$l_{clf}=-\sum_{c=0}^{C-1}y_{c}\log F(G\left(L_{1},\ldots ,L_{k}\right))$$

where $L_{1},\ldots ,L_{k}$ are the low-resolution video frames, *C* and $y_{c}$ are the number of action class and the labels of action, respectively.

### 3.3. Training Strategy

#### 3.3.1. Data Augmentation

Prior works on low-resolution recognition mainly performed experiments on two standard datasets, i.e., HMDB51 [20] and UCF101 [19], which have only 3.7 k and 0.2 k training videos, respectively. The scale of the two datasets is truly small. Since the similarity of adjacent frames in videos is extraordinarily high, it makes no sense to use all of the frames in a video for training our super-resolution module. Compared with other similar low-resolution tasks [7,8] using GANs, such as SOD-MTGAN [7], which has 80k training images, the amount of data our super-resolution module can use is much smaller. It is risky to train with such a limited amount of data as it can easily cause overfitting.

Motivated by the practice in [1], data augmentation is employed for training our proposed GAN. In the learning phase of the GAN for HMDB51, the UCF101 dataset is introduced. Different from modifying the architecture of our network, we directly merge two datasets. Specifically, we first divide the two datasets into training and test sets according to the official partition file. Then, the training sets of HMDB51 and UCF101 are merged to train the super-resolution module.

#### 3.3.2. Long-Range Temporal Convolutions

Previous works for high-resolution activity recognition with CNN architectures, such as C3D [2] and R2 + 1D [22], typically learned activity representations at the level of a few video frames and thus failed to model longer-range temporal information. Despite this minor flaw, these methods have a powerful performance due to the abundant spatial information in high-resolution videos. However, for extreme low-resolution videos, the spatial information of a single frame is limited. Following the idea of [31], we use long-term temporal convolutions to model spatial–temporal information over a longer range to better learn low-resolution video representations. Specifically, the number of input frames is typically 16. We boost the number to 64, which can cover a more complete temporal extent to operate spatial–temporal convolutions.

## 4. Experiments

### 4.1. Dataset

The HMDB51 [20] and Dogcentric [32] datasets have been popularly used for extreme low-resolution recognition evaluation in previous works [3,4,5,23]. We choose the HMDB51 dataset to make a direct comparison between our approach and previous works. The UCF101 [19] dataset is chosen instead of Dogcentric for the following reasons. On the one hand, our goal is to recognize reliable human, not dog, activities at distances and to preserve human privacy in extreme low-resolution videos. The videos in Dogcentric are taken from the dog’s viewpoint and record the dog’s activities, such as turning the dog’s head to the right/left and playing with a ball. On the other hand, UCF101 contains various videos ranging from videos in which humans near the camera to videos in which humans are poorly visible in the wild, which fits our goal effectively. All these factors make UCF101 a more reasonable and challenging dataset for extremely low-resolution activity recognition.

**Figure 7:**
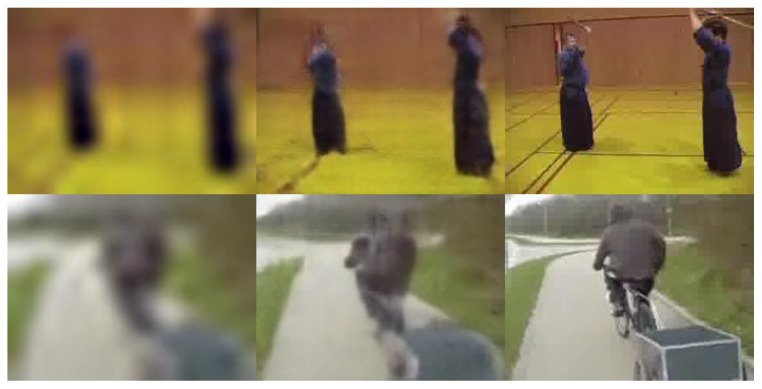
Visualization of some corresponding low-, super-, and high-resolution images of HMDB51. The left column shows low-resolution images; the middle column shows super-resolved images; the right column shows high-resolution images.

Specifically, HMDB51 consists of 6766 video clips that are collected from movies and web videos with 51 activity categories. UCF101 is a popular video dataset containing 13,320 video clips belonging to 101 activity classes. The resolution of the above two datasets is 240 × 320 pixels. To simulate an extremely low-resolution dataset, we resize these videos to 12 × 16 pixels with average downsampling and then resize the 12 × 16 videos back to their original size using bicubic interpolation. Several corresponding low- and high-resolution frames are shown in Figure 7. Then, these datasets are split into two parts via the provided train/test split files.

### 4.2. Implementation Details

Our training process consists of two stages: (1) training the super-resolution module and recovering super-resolution frames from low-resolution video and (2) training the recognition module with the recovered frames of each video as inputs.

For the super-resolution module, we train our GAN on the HMDB51 and UCF101 datasets at low resolution from scratch. As discussed before, we use simulated low-resolution data as inputs and high-resolution data as labels. Adam [33] is adopted to optimize the network parameters with a learning rate of $10^{-3}$ and a weight decay of $10^{-5}$. The whole process stops at 300 epochs, with the batch size set to 60.

For the recognition module, we follow [30]. Using their available pretrained model, we finetune it on the HMDB51 and UCF101 datasets at low resolution. We adopt 16/64 frames as inputs, respectively. Stochastic gradient descent [34] is employed to optimize the network parameters with a learning rate of $10^{-3}$ and a weight decay of $10^{-5}$. All our experiments are implemented in PyTorch on Ubuntu with two Nvidia 1080Ti GPUs.

### 4.3. Ablation Studies

**Influence of the Super-Resolution Module.**Table 2 (the 1st row vs. 2nd row and 3rd row vs. 4th row) compares the performance of our model with/without the super-resolution module. From Table 2, it is observed that without long-range temporal convolutions but with the enhancement of our super-resolution module, the performance of our model outperforms other methods without the super-resolution module by a small margin (i.e., 0.6% accuracy on HMDB51 and 1% accuracy on UCF101). After long-range temporal convolutions are introduced, the influence of the super-resolution module increases. The performance of our model with the super-resolution module outperforms other methods without that module by a sizable margin (i.e., 1.2% in accuracy on HMDB51 and 1.6% on UCF101). Figure 7 shows the corresponding low- and high-resolution frames and super-resolved frames recovered from the super-resolution module. These results demonstrate that the super-resolution module can effectively help increase the accuracy of low-resolution activity recognition. With long-range temporal convolutions, the lost information recovered from low-resolution frames can be more fully explored. In addition, as shown in Table 3, our approach obtains considerable performance on TinyVIRAT dataset which makes a margin of about 1% comparing with baseline model(the 1st row vs. 2nd row and 3rd row vs. 4th row).

**Influence of Long-Range Temporal Convolutions.** From Table 2 (1st row vs. 3rd row and 2nd vs. 4th row), we can see that the accuracy drops by 7.5% and 8.1%, respectively, without long-range temporal convolutions, and from Table 3 (1st row vs. 3rd row and 2nd vs. 4th row), we can see that the accuracy drops by 5.2% and 5.1%, respectively, without long-range temporal convolutions. The reason is that without long-range temporal convolution, we can only model a limited amount of the temporal information which is important for activity recognition. To effectively learn spatial–temporal information in low-resolution videos, we use long-range temporal convolutions to train our network.

**Figure 8:**
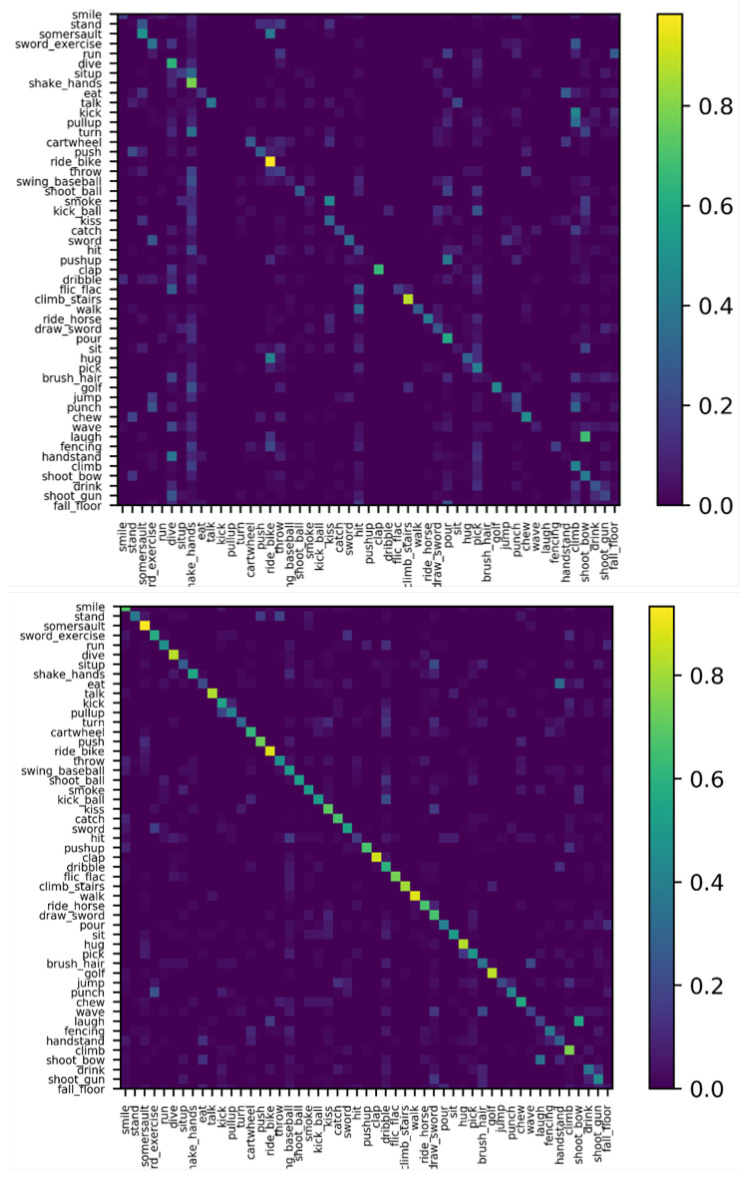
Confusion matrix on the 12 × 16 HMDB51 dataset. The X-axis denotes the predicted labels, and the y-axis presents the ground truth labels. (**a**) The result of our baseline model without the super-resolution module and long-range temporal convolutions. (**b**) The result of our proposed model with a super-resolution module and long-range temporal convolutions.

**Figure 9:**
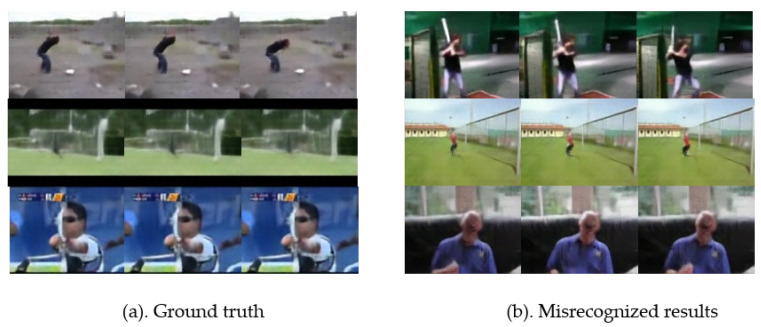
Snapshots of parts of misrecognized activities. (**a**) is the ground truth label, and (**b**) is the misrecognized activity.

**Evaluation of Our Method.** As shown in Figure 8, the confusion matrices illustrate that the performance of our proposed model with the super-resolution module and long-range temporal convolutions is visually more remarkable than that of our baseline method. Figure 8b shows that the recognition accuracy of most activities is considerably high. However, several actions, such as ‘hit’, ‘jump’, and ‘shoot bow’ are misrecognized as ‘swing baseball’, ‘catch’, and ‘laugh’. This is because these actions have similar subactions and lose too much information in the extreme low-resolution videos, which is demonstrated in Figure 9.

### 4.4. State-of-the-Art Comparison

We compare our proposed method with several state-of-the-art low-resolution activity recognition models [3,4,5,35] on the challenging 12 × 16 HMDB51 dataset. Table 4 lists the performance, modalities and number of input frames, from which we conclude that our method outperforms all other state-of-the-art methods on the HMDB51 dataset. More importantly, in the case where only 16-RGB frames are used as input, our method still obtains better performance than the second-best low-resolution recognition model by approximately 1.5%. If we follow the set of input frames of previous works, our method outperforms the second best model by a large margin. Moreover, we make a comparison on UCF101 dataset between our proposed method and DVSR. Table 5 shows the comparing result from which we can see our approach outperforms DVSR [9] by a considerable margin of accuracy. This clearly demonstrates the effectiveness of our method on low-resolution activity recognition.

## 5. Discussion and Conclusions

We must mention that in this paper, our goal of using GAN is to generate super-resolution images from low-resolution images to help recognition. It is true that we can use more advanced variants of GAN to obtain better super-resolution performance, but we restrict our choice to SDSR based on two factors: (1) the basic idea of this manuscript is to propose a framework for extreme low-resolution activity recognition, not a new SR method; and (2) for activity recognition, it is not necessary to recover all the details but general silhouettes of humans and objects. It must also be mentioned that different SR modules may further improve the subsequent recognition performance, and this is planned for our future work.

In this paper, we propose a super-resolution generative network-based method to recognize activities in extreme low-resolution videos. Our method consists of two modules, namely, a super-resolution module and an activity recognition module. The proposed super-resolution module generates super-resolution frames from low-resolution frames, which can recover lost information to improve recognition. The recognition module adopts the recovered frames as inputs and predicts the category of the activity in the low-resolution videos. Extensive experiments on the HMDB51 and UCF101 datasets demonstrate that our method improves the state-of-the-art accuracy performance compared to other methods.

In our future work, more network architectures for both super-resolution and activity recognition will be evaluated. In addition, more datasets with multiple levels of resolution will be included to evaluate the robustness of the proposed model.

## Figures and Tables

**Table 1:** The architecture of activity feature extractor. F is the number of feature channels corresponding in Figure 6, and N is the number of blocks in each layer.

Part	Output Size	F	N
conv1	8 × 56 × 56	64	conv 7 × 7 × 7, 64
conv2_x	8 × 56 × 56	128	8
conv3_x	4 × 28 × 28	256	24
conv4_x	2 × 14 × 14	512	36
conv5_x	1 × 7 × 7	1024	3

**Table 2:** Performance of our proposed method with/without two mechanisms on HMDB51 and UCF101.

Super-Resolution	Long-Range	Accuracy	
Module	Temporal Convolutions	HMDB51	UCf101
×	×	45.8%	65.3%
√	×	46.6%	66.2%
×	√	53.3%	69.6%
√	√	54.6%	71.1%

**Table 3:** Performance of our proposed method with/without two mechanisms on TinyVIRAT.

Super-Resolution	Long-Range Temporal	F1 Score
Module	Convoluton Module	TinyVIRAT
×	×	73.89%
√	×	74.11%
×	√	79.02%
√	√	79.77%

**Table 4:** The performance of the proposed method and other state-of-the-art methods on the 12 × 16 HMDB51 dataset.

Methods	Modalities	Input Frames	Accuracy
pLRN + Tennet [35]	RGB	-	21.7%
ISR [23]	RGB	-	28.68%
Semi-Coupled [3]	RGB + Optical flow	64	29.2%
Multi-Siamese [5]	RGB + Optical flow	64	37.7%
DVSR [9]	RGB	16	41.63%
Fully-Coupled [4]	RGB + Optical flow	64	44.96%
Ours	RGB	16	46.4%
Ours	RGB	64	54.4%

**Table 5:** The performance of the proposed method and other state-of-the-art methods on the UCF101 dataset. LRTC denotes long-range temporal convolutons.

Method	Input Size	Accuracy
Bicubic I3D	14 × 14	14.1%
DVSR	14 × 14	68.2%
Prog.DVSR	14 × 14	70.6%
Ours	12 × 16	66.2%
Ours + LRTC.	12 × 16	71.1%
